# Identification of NEO1 as a prognostic biomarker and its effects on the progression of colorectal cancer

**DOI:** 10.1186/s12935-020-01604-1

**Published:** 2020-10-17

**Authors:** Meng Zhang, Zhou Zhou, Xue-kai Pan, Yun-jiao Zhou, Hai-ou Li, Pei-shan Qiu, Meng-na Zhang, Ru-yi Peng, Hai-zhou Wang, Lan Liu, Jing Liu, Qiu Zhao

**Affiliations:** 1 Department of Gastroenterology, Zhongnan Hospital of Wuhan University, No. 169, Donghu Road, Wuchang District, Wuhan, 430071 Hubei Province China; 2 Hubei Clinical Center & Key Lab of Intestinal & Colorectal Diseases, Wuhan, 430071 China; 3 Department of Gastroenterology and Hepatology , Leiden University Medical Center, Leiden, The Netherlands

**Keywords:** Biomarker, Colorectal cancer, Neogenin-1, Prognosis

## Abstract

**Background:**

Due to the high morbidity and poor clinical outcomes, early predictive and prognostic biomarker identification is desiderated in colorectal cancer (CRC). As a homologue of the Deleted in Colorectal Cancer (DCC) gene, the role of Neogenin-1 (NEO1) in CRC remained unveiled. This study was designed to probe into the effects and potential function of NEO1 in CRC.

**Methods:**

Online databases, Gene Set Enrichment Analysis (GSEA), quantitative real-time PCR and western blotting were used to evaluate NEO1 expression in colorectal cancer tissues. Survival analysis was performed to predict the prognosis of CRC patients based on NEO1 expression level. Then, cell proliferation was detected by colony formation and Cell Counting Kit 8 (CCK-8) assays. CRC cell migration and invasion were examined by transwell assays. Finally, we utilized the Gene Set Variation Analysis (GSVA) and GSEA to dig the potential mechanisms of NEO1 in CRC.

**Results:**

Oncomine database and The Cancer Genome Atlas (TCGA) database showed that NEO1 was down-regulated in CRC. Further results validated that NEO1 mRNA and protein expression were both significantly lower in CRC tumor tissues than in the adjacent tissues in our clinical samples. NEO1 expression was decreased with the progression of CRC. Survival and other clinical characteristic analyses exhibited that low NEO1 expression was related with poor prognosis. A gain-of-function study showed that overexpression of NEO1 restrained proliferation, migration and invasion of CRC cells while a loss-of-function showed the opposite effects. Finally, functional pathway enrichment analysis revealed that NEO1 low expression samples were enriched in inflammation-related signaling pathways, EMT and angiogenesis.

**Conclusion:**

A tumor suppressor gene NEO1 was identified and verified to be correlated with the prognosis and progression of CRC, which could serve as a prognostic biomarker for CRC patients.

## Background

The morbidity of colorectal cancer (CRC) is rising sharply in those who are younger than 50 years old. What worries us more is that CRC has ranked the first causes of cancer death in men age 20–49 during 2012 to 2016 [1–3]. Although the 5-year survival rate for patients with CRC has ascended to about 65%, the rate declines to 12% of patients diagnosed with stage IV, emphasizing the urgently need to identify early predictive and prognostic biomarkers [4, 5]. What’s more, it has been clearly recognized that CRC is a heterogeneous disease, which demonstrating that either inter-tumor or intra-tumor showed diverse molecular traits, resulting in distinct clinical outcomes [6]. Recently, bioinformatics methods are widely used to analyze the high throughput sequencing data and microarray data for diseased related gene prediction [7]. For example, the transcriptome and DNA methylome analyses were utilized to reveal increased CRC risk in obesity [8]. KRAS, p53 and SMAD4 were identified as potential biomarkers to evaluate prognosis and metastasis for patients with CRC by analyzing TCGA datasets [9]. With the advent of new technologies, like genome-wide expression profiling studies and RNA sequencing, tens of thousands of differentially expressed genes (DEGs) in CRC have been uncovered [10–12]. Whether these DEGs could be valid biomarkers and the exact mechanisms in CRC remained to be studied.

Neogenin-1 (NEO1) was originally identified as a homologue of the Deleted in Colorectal Cancer (DCC) gene, which acted as a receptor for Netrins and Repulsive Guidance Molecule (RGM) proteins [13]. RGMa/Netrin-1/NEO1 signaling was proved to relate to retinal ganglion cell axon guidance and dorsoventral patterning in the embryonic forebrain [14]. Besides, NEO1 plays vital roles in apoptosis, differentiation, adhesion and migration [15]. Recently, abnormal expression of NEO1 has been demonstrated in some kinds of cancer such as glioma, breast cancer and pancreatic cancer [16–18], but little is known about its specific functions. A study has identified that RGMA and its receptor NEO1 were both down-regulated in most CRCs and adenomas. Results further demonstrated that RGMA overexpression in CRC cells could suppress cell proliferation, migration, and invasion while increase apoptosis to play as a tumor suppressor in CRC [13]. However, the specific role of its receptor NEO1 in CRC needs further exploration. This study was designed to probe into the effects and potential function of NEO1 in CRC.

Here, the relationship between NEO1 expression level and prognosis of CRC was explored. By transfection NEO1 plasmids or siRNAs, the effects of NEO1 in CRC cells were evaluated in vitro. Finally, the potential mechanism of NEO1 in CRC was tested. In total, our findings demonstrated that NEO1 could be a prognostic biomarker and regulate tumor progression in CRC.

## Materials and methods

### Data collection and preprocessing

The human colonic neoplasm mRNA expressing data were downloaded from Gene Expression Omnibus (GEO) database (https://www.ncbi.nlm.nih.gov/geo/). Meanwhile, CRC RNA-sequencing data from The Cancer Genome Atlas (TCGA) database (https://genome-cancer.ucsc.edu/) with intact clinic features were obtained for further validation. After Robust Multiarray Averaging (RMA) background correction, log_2_ transformation and quantile normalization, the “affy” R package was used for median-polish probe set summarizing. Sample clustering based on the interval between diverse samples in average link was used to assess microarray quality.

## Clinical samples

Clinical features from colon cancer datasets GSE41258 (n = 202) and GSE39582 (n = 585) were extracted (Tables 1, 2). Fifty-three cases of clinical CRC specimens and paired non-tumor tissues were collected from Zhongnan Hospital of Wuhan University (Wuhan, China) and diagnosed by the Pathology Department (Table 3). The written informed consent was obtained from the patients. This program was admitted by the ethics committee of Zhongnan Hospital of Wuhan University (protocol #2,017,012, #2,017,014).

**Table 1:** Characteristics of patients with colon cancer in GSE41258

Clinical characteristics	NEO1 expression			Chi-square	P value
	Total	High	Low		
Gender					
Male	106	50	56	0.715	0.398
Female	96	51	45		
Age					
< 65	87	46	41	0.505	0.477
≥65	115	55	60		
T stage					
T1-T2	40	27	13	6.11	0.013*
T3-T4	162	74	88		
N stage					
N0	101	50	51	0.02	0.888
N1-N2	101	51	50		
M stage					
M0	138	77	61	5.855	0.016*
M1	64	24	40		
Clinical stage					
I	30	21	9	5.637	0.018*
II–IV	172	80	92		
Recurrence					
Yes	39	13	26	9.135	0.003**
No	116	71	45		
Microsattelite instability					
pMMR	145	66	79	3.749	0.053
dMMR	38	24	14		
p53 mutation status					
Mutant	99	49	50	0.319	0.572
Wild-type	58	26	32		

^*^P < 0.05, **P < 0.01

**Table 2:** Characteristics of patients with colon cancer in GSE39582

Clinical characteristics	NEO1 expression			Chi-square	*P* value
	Total	High	Low		
Gender					
Male	322	165	157	0.383	0.536
Female	263	128	135		
Age					
< 65	216	106	110	0.165	0.685
≥ 65	368	187	181		
T stage					
Tis-T2	65	38	27	10.955	0.000074***
T3-T4	498	166	332		
N stage					
N0	314	180	134	14.332	0.000153***
N1-N3	243	100	143		
M stage					
M0	499	262	237	7.107	0.008**
M1	61	21	40		
Clinical stage					
0-II	313	179	134	14.305	0.000155***
III-IV	270	112	158		
Tumor location					
Proximal	232	104	128	3.988	0.046*
Distal	351	187	164		
Chemotherapy.adjuvant					
Yes	240	115	125	1.467	0.226
NO	326	173	153		
mmr.status					
dMMR	77	36	41	0.471	0.492
pMMR	459	234	225		
Cimp.status					
Positive	93	34	59	8.09	0.004**
Negative	420	222	198		
Cin.status					
Positive	369	191	178	0.029	0.865
Negative	112	59	53		
tp53.mutation					
Mutant	190	78	112	5.899	0.015*
Wild type	161	87	74		
Kras.mutation					
Mutant	217	107	110	0.16	0.689
Wild type	328	156	172		
Braf.mutation					
Wild type	461	230	231		
Mutant	51	16	35	6.309	0.012*

^*^P < 0.05, **P < 0.01, ***P < 0.001

**Table 3:** Characteristics of patients with colorectal cancer in Zhongnan Hospital

Clinical characteristics	NEO1 expression			Chi-square	*P* value
	Total	High	Low		
Gender					
Male	36	20	16	0.955	0.328
Female	17	7	10		
Age					
≥ 65	24	12	12	0.016	0.901
< 65	29	15	14		
T stage					
2	2	2	0	9.558	0.003**
3	38	23	15		
4	13	2	11		
N stage					
0	29	17	12	2	0.623
1	15	7	8		
2	7	3	4		
3	1	0	1		
M stage					
0	48	27	21	5.733	0.023*
1	5	0	5		
Clinical stage					
1	2	2	0	7.11	0.048*
2	26	15	11		
3	20	10	10		
4	5	0	5		
Histology					
Ad	33	17	16	2.992	0.406
Tubular Ad	11	6	5		
Villoglandular Ad	2	2	0		
Mucinous Ad	7	2	5		
Differentiation					
Moderate-High	1	0	1	4.068	0.207
Moderate	32	20	12		
Moderate-Low	10	5	5		
Low	2	0	2		

*Ad* Adenocarcinoma

*P < 0.05, **P < 0.01

### Cell culture

Human colon cancer cell lines (HCT116, DLD1, SW480) and human colonic epithelial cell line (NCM460) were purchased from China Center for Type Culture Collection (CTCC, Wuhan, China). All cells were cultured in DMEM (HyClone, USA) with 10% fetal bovine serum (Gibco, Australia) at 37 °C with 5% CO_2_.

### Plasmids and siRNA transfection

PCMV3-NEO1 plasmids were purchased from Sino Biological (Beijing, China). NEO1 siRNAs were synthesized by Ribobio (Guangzhou, China). Using Lipofectamine 2000 (Invitrogen, USA), pCMV3-NEO1 plasmids (2.5 μg each well) were transfected to DLD1 and HCT116 cells to overexpress NEO1 expression following the manufacturer’s protocol. Empty vector pCMV3 plasmids were used as control. NEO1 siRNAs (siNEO1 #1: GACCAAAGGTCGAAGATCA, siNEO1 #2: GAGCTGTCTATGACCGATA) were transfected to SW480 cells to knockdown NEO1 expression. Scrambled siRNAs were used as negative control.

### RNA preparation and quantitative real-time PCR (qPCR)

RNA from human colonic epithelial cell line (NCM460), human colon cancer cell lines (HCT116, DLD1, SW480), colorectal cancer tissues and para-carcinoma tissues was isolated by Trizol reagent (Invitrogen, USA) based on the manufacturer’s protocols. 1 μg RNA was used to synthesize cDNA by RevertAid First Strand cDNA Synthesis Kit (Thermo Scientific, USA). Quantitative real-time PCR (qPCR) was subsequently performed using ABI QuantStudio™ 6 Flex System (USA) with SYBR® Premix Ex TaqTM II mix (Takara, Japan). Gene primers were as follows: NEO1 (Forward 5′-TGGGTTATTGAGCCTGTTG-3′ and Reverse 5′-GGAGTCCGCTTTAGGTGTTC-3′); GAPDH (Forward 5′-GTCTCCTCTGACTTCAACAGCG-3′ and Reverse 5′-ACCACCCTGTTGCTGTAGCCAA-3′). The relative mRNA expression levels were calculated using the 2^−△△Ct^ method against GAPDH. Data for NCM460 and para-carcinoma tissue were used as controls respectively.

### Western blotting analysis

CRC cell protein was collected using NP40 Lysis buffer (Beyotime, China) and its concentration was examined by BCA kits (Beyotime, China). 30 μg protein of each group was loaded on 8% SDS-PAGE gel, transferred to PVDF membranes (Millipore, USA) and then blocked with 5% fat-free milk. Incubating the membranes all night at 4 °C in the primary antibodies: NEO1 (ab183511, Abcam, UK), GAPDH (GB13002, Servicebio, China). Anti-rabbit secondary antibody (GB23303, Servicebio, China) was further applied. The band detection was performed by Enhanced Chemi-luminescence reagents (Thermo, USA).

### Cell growth assay

Cell proliferation was tested by colony formation and Cell Counting Kit 8 (CCK-8) assays. For colony formation, 1000 cells were seeded in 6-well plates with 3 ml medium per well. Groups were divided by transfecting corresponding plasmids (2.5 μg per well) or siRNAs (100 pmol per well) utilizing Lipofectamine 2000. Then, they were cultured for 10 days. After 4% paraformaldehyde fixing and 0.1% crystal violet staining, colonies with over 50 cells were calculated. For CCK-8 assays, colon cancer cells were seeded at 3000 cells per well in a 96-well plate. Plasmids (100 ng per well) or siRNAs (4 pmol per well) were transfected by Lipofectamine 2000 into different groups. After incubating at 37 °C for 0, 1, 2 and 3 days, CCK-8 solution was added in tested wells and incubated for 2 h. Finally, OD_450_ was detected by a microplate reader (BioTek ELx800, USA).

### Transwell assay

Boyden chambers (Corning, USA) with an 8 μm pore size membrane were utilized for transwell migration and invasion assay. The upper chamber was pre-coated with Matrigel (BD, USA) for invasion assay. 2 × 10^5^ transfected or control cells were seeded in the upper with 200 μl serum-free medium while 20% FBS medium was added into the lower. After 24-h incubation at 37 °C, cells were fixed and stained by crystal violet for 25 min. Then stained cells were observed and calculated under an optical microscope.

### Functional enrichment analysis

Keyword “colon” was searched on Gene Set Enrichment Anaylsis (GSEA) (https://software.broadinstitute.org/gsea/msigdb/search.jsp). Download all gene sets. Biological process enriched in NEO1 was explored by GSEA. The Gene Set Variation Analysis (GSVA) method from the “GSVA” R package was used to investigate the significantly altered pathways between high and low NEO1 expression in GSE39582.

### Statistical analysis

CRC samples were categorized into NEO1 low expression and high expression group according to the NEO1 expression median of GSE41258, GSE39582 and Zhongnan Hospital tissues. The strength of relationship between the NEO1 expression and the CRC patient clinical features was estimated by Chi-square analysis. Survival analyses were performed by Kaplan–Meier methods and compared by the log-rank test. In vitro experiments, all tests were performed at least three times and data were shown as mean ± s.e.m. GraphPad PRISM software was used to make graphs and analyze results. The statistical significance was evaluated by two-tailed Student’s t-tests. *P* < *0.05* was considered statistical significance.

## Results

### NEO1 expression is down-regulated in CRC tumor tissues

**Fig. 1:**
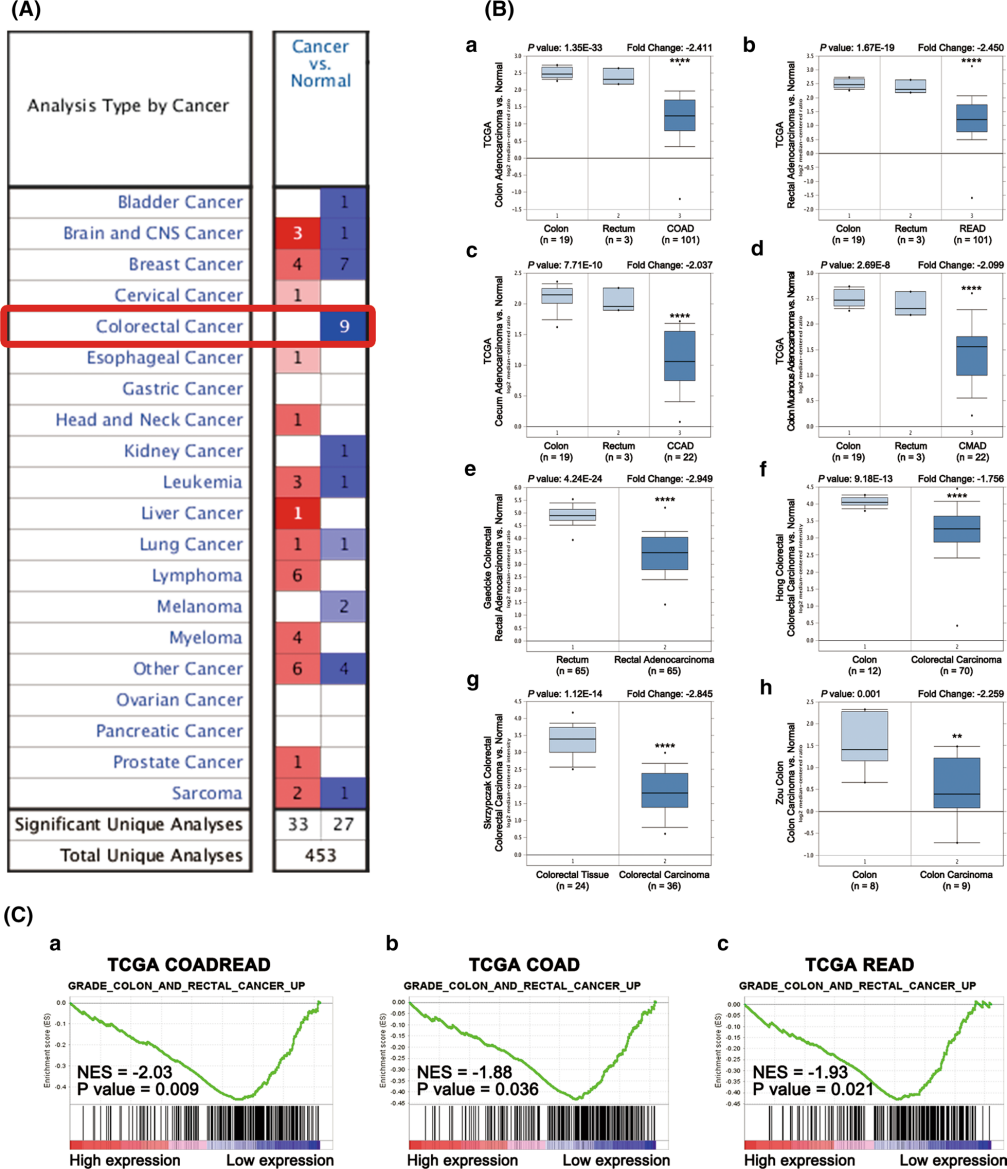
Online dataset analysis revealed NEO1 specific expression in colorectal cancer (CRC). (**A**) Oncomine database showed that NEO1 was down-regulated in CRC, compared with normal tissues. The red represented that NEO1 was up-regulated while the blue meant down-regulated. (**B**) NEO1 expression in CRC was decreased in TCGA dataset (a-d) and other four online datasets (e-h). (**C**) Gene set enrichment analysis (GSEA) indicated that NEO1 was down-regulated in both rectal and colon carcinoma compared to normal mucosa samples. **P<0.01, ****P<0.0001; compared with normal tissues

An online oncogene microarray database Oncomine (https://www.oncomine.org) was used to identify the differential mRNA expression of NEO1 between cancers and normal tissues. Among total 453 analyses, 60 analyses were considered significant (p-value ≤ 0.05; fold change ≥ 1.5; gene rank top ≤ 10%). In detail, 33 analyses, which involved in Brain and CNS cancer, Breast cancer, Lymphoma, Myeloma and many other kinds of cancers, showed statistically significant higher NEO1 mRNA expression levels in tumors, while 27 analyses, such as in Bladder cancer, Colorectal cancer and Kidney cancer, showed lower expression. Especially, all analyses (9 analyses) about colorectal cancer exhibited coincidentally lower NEO1 expression in tumors (Fig. 1a).

Furthermore, in TCGA dataset, the mRNA level of NEO1 was significantly decreased in colon adenocarcinoma (COAD) (fold change = -−2.411, *p* = 1.35E-33), rectal adenocarcinoma (READ) (fold change = -−2.450, *p* = 1.67E-19), cecum adenocarcinoma (CCAD) (fold change = -−2.037, *p* = 7.71E-10) and colon mucinous adenocarcinoma (CMAD) (fold change = -−2.099, *p* = 2.69E-8) (Fig. 1B(a-d)). Meanwhile, NEO1 also showed lower expression in rectal adenocarcinoma in Gaedcke dataset (fold change = -−2.949, *p* = 4.24E-24), in colorectal carcinoma in Hong dataset (fold change = -−1.756, *p* = 9.18E-13) and Skrzypczak dataset (fold change = -−2. 845, *p* = 1.12E-14), and in colon carcinoma in Zou dataset (fold change = -−2.259, *p* = 0.001) (Fig. 1B(e-h))). GSEA demonstrated that NEO1 was down-regulated in both rectal and colon carcinoma compared to normal mucosa samples by using TCGA COADREAD, TCGA COAD and TCGA READ (Fig. 1C(a-c)).

**Fig. 2:**
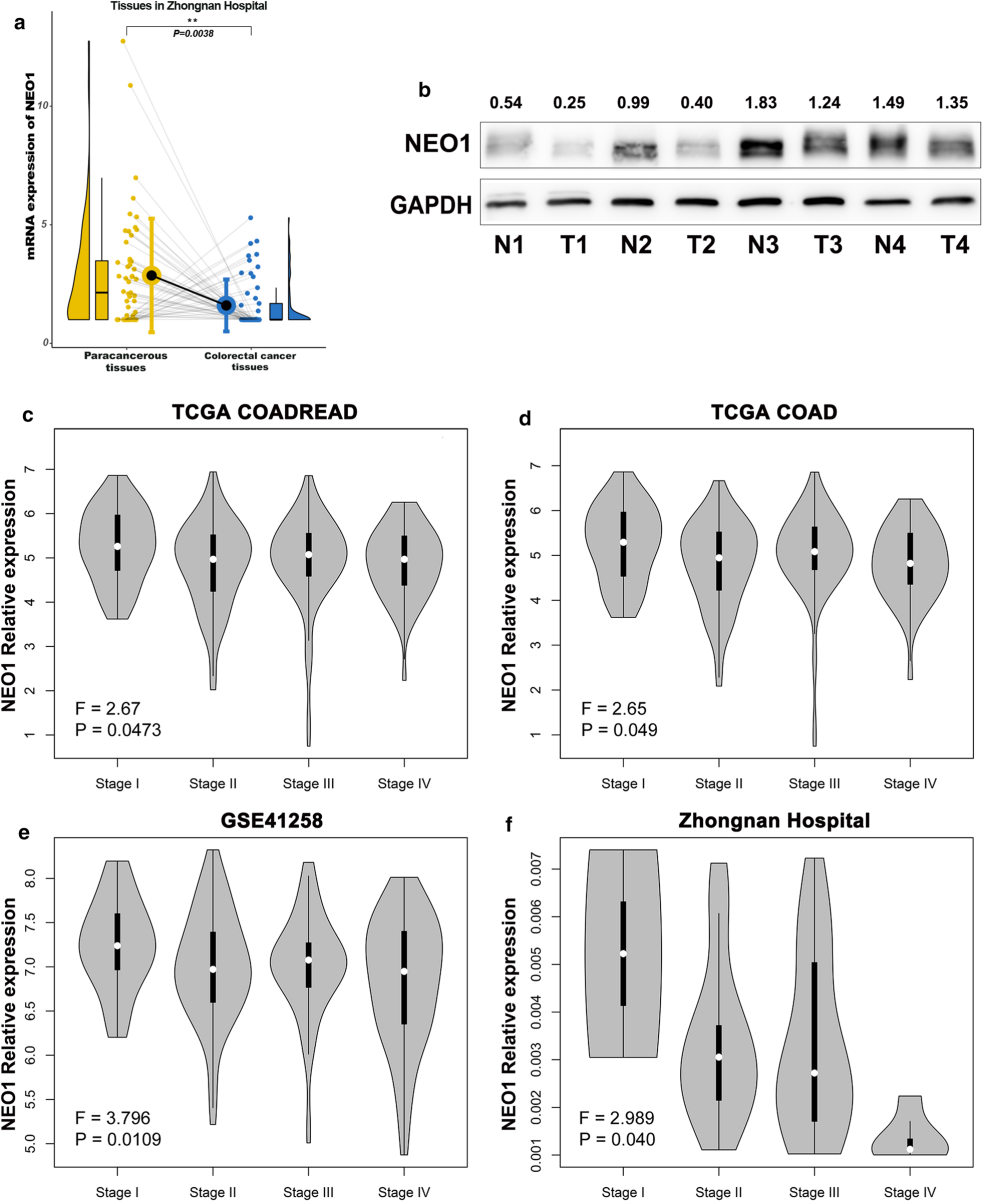
NEO1 was down-regulated in CRC patients. **a** Quantitative real-time PCR analysis showed that NEO1 was down-regulated in 53 CRC tissues compared to paired adjacent tissues (p = 0.0038). **b** Western blotting analysis demonstrated that NEO1 was down-regulated in 4 CRC tissues. The gray intensity of protein expression was quantified by image J software. NEO1 protein expression value was calculated against GAPDH. **c-f** The expression of NEO1 in CRC was negatively associated with the tumor stage in TCGA (**c**, **d**), GSE41258 datasets (**e**) and Zhongnan Hospital tissues (**f**). **P < 0.01

To further validate the expression level of NEO1, we collected 53 pairs of tumor tissues and adjacent non-tumor tissues in Zhongnan Hospital of Wuhan University. The results confirmed that NEO1 was down-regulated in CRC tumor tissues when compared with non-tumor tissues, both in mRNA level (p = 0.0038, n = 53) (Fig. 2a) and protein level (Fig. 2b). Furthermore, according to Gene Expression Profiling Interactive Analysis (GEPIA) database (https://gepia.cancer-pku.cn/), the expression of NEO1 was decreased with the progression of colorectal cancer and colon cancer (Fig. 2c, d). Consistently, in GSE41258 dataset and Zhongnan Hospital tissues, the expression of NEO1 was proved to be significantly and negatively associated with the tumor stage for CRC (Fig. 2e, f). The above data sufficiently demonstrated that NEO1 expression was down-regulated in CRC and further decreased with the progression of CRC.

### Low expression of NEO1 predicts poor prognosis of CRC patients

**Fig. 3:**
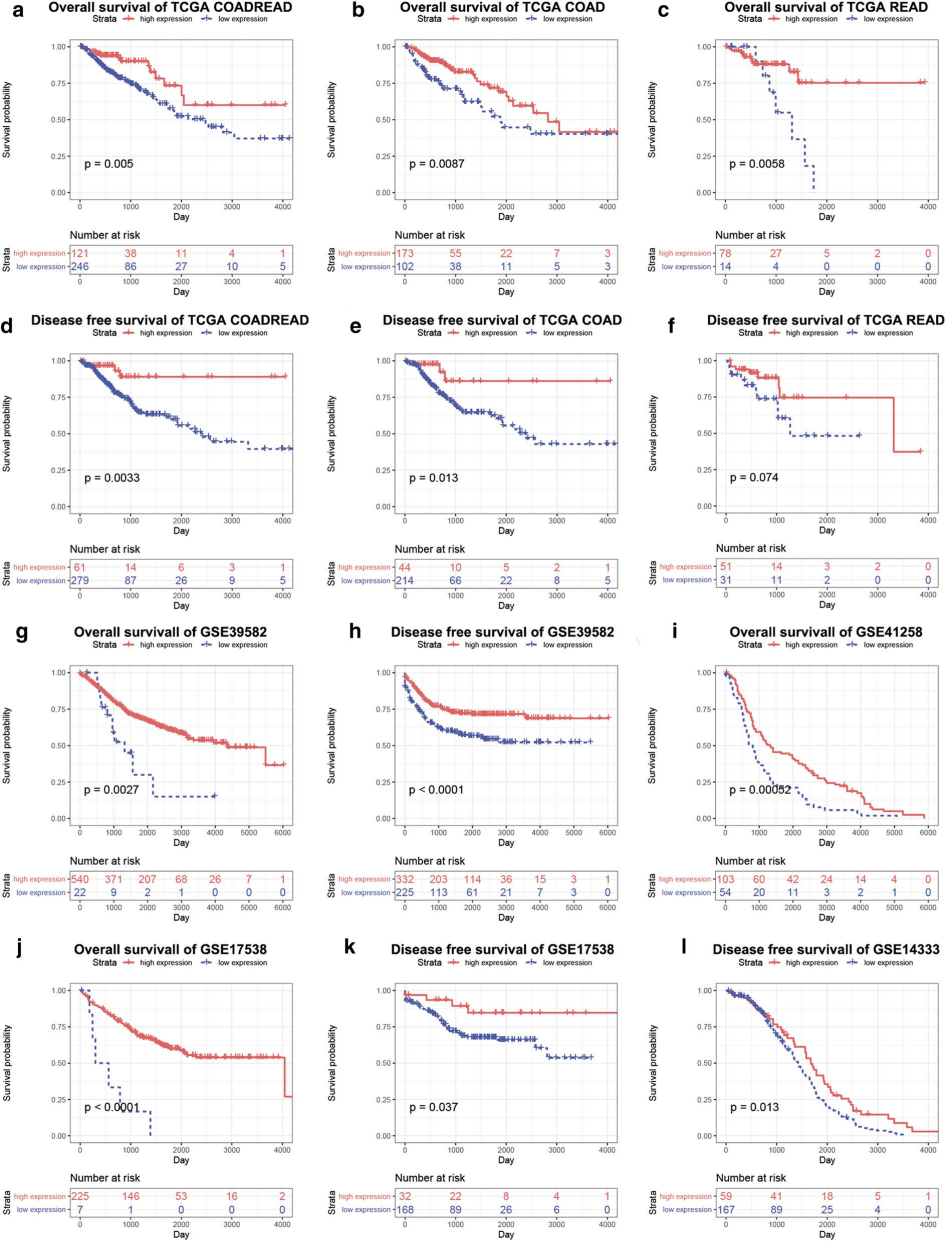
CRC patient with low expression level of NEO1 had a significantly shorter survival. **a**, **d** Overall survival (**a**) and disease free survival (**d**) of TCGA COADREAD dataset. **b**, **e** Overall survival (**b**) and disease free survival (**e**) of TCGA COAD dataset. **c**, **f** Overall survival (C) and disease free survival (**f**) of TCGA READ dataset. **g**, **h** Overall survival (**g**) and disease free survival (**h**) of GSE39582 dataset.** I** Overall survival of GSE41258 dataset. **J**, **K** Overall survival (**j**) and disease free survival (**k**) of GSE17538 dataset. **l** Disease free survival of GSE14333 dataset

To further explore the role of NEO1 in CRC, the overall survival (OS) and disease free survival (DFS) analyses were performed. In TCGA dataset, decreased expression of NEO1 had a significantly shorter OS on COADREAD (p = 0.005), COAD (p = 0.0087) and READ (p = 0.0058) (Fig. 3a-c). Consistent with the OS analyses, the DFS analyses exhibited the same tendency in COADREAD (p = 0.0033) and COAD (p = 0.013), but not in READ (p = 0.074) (Fig. 3d-f). Survival analysis were further confirmed in GSE39582 (p = 0.0027 in OS and p < 0.0001 in DFS), GSE41258 (p = 0.00052 in OS), GSE17538 (p < 0.0001 in OS and 0.037 in DFS) and GSE14333 datasets (p = 0.013 in DFS) (Fig. 3g-l).

Next, the relationship between NEO1 expression and the clinical features of CRC patients was studied. As shown in Table 1, colon cancer patients in NEO1 low expression group were associated with worse T stage (p = 0.013), M stage (p = 0.016) and recurrence (p = 0.003). In GSE39582, patients with high expression were related to better T stage (p = 0.000074), N stage (p = 0.000153), M stage (p = 0.008), clinical stage (p = 0.000155) and cimp status (p = 0.004) (Table 2). Furthermore, in Zhongnan Hospital CRC tumor tissues, lower expression of NEO1 was correlated with worse T stage (p = 0.003), M stage (p = 0.023) and clinical stage (p = 0.048) (Table 3). The above data showed that lower NEO1 expression was correlated with poorer prognosis.

### NEO1 regulates cell proliferation, migration and invasion in CRC cells

**Fig. 4:**
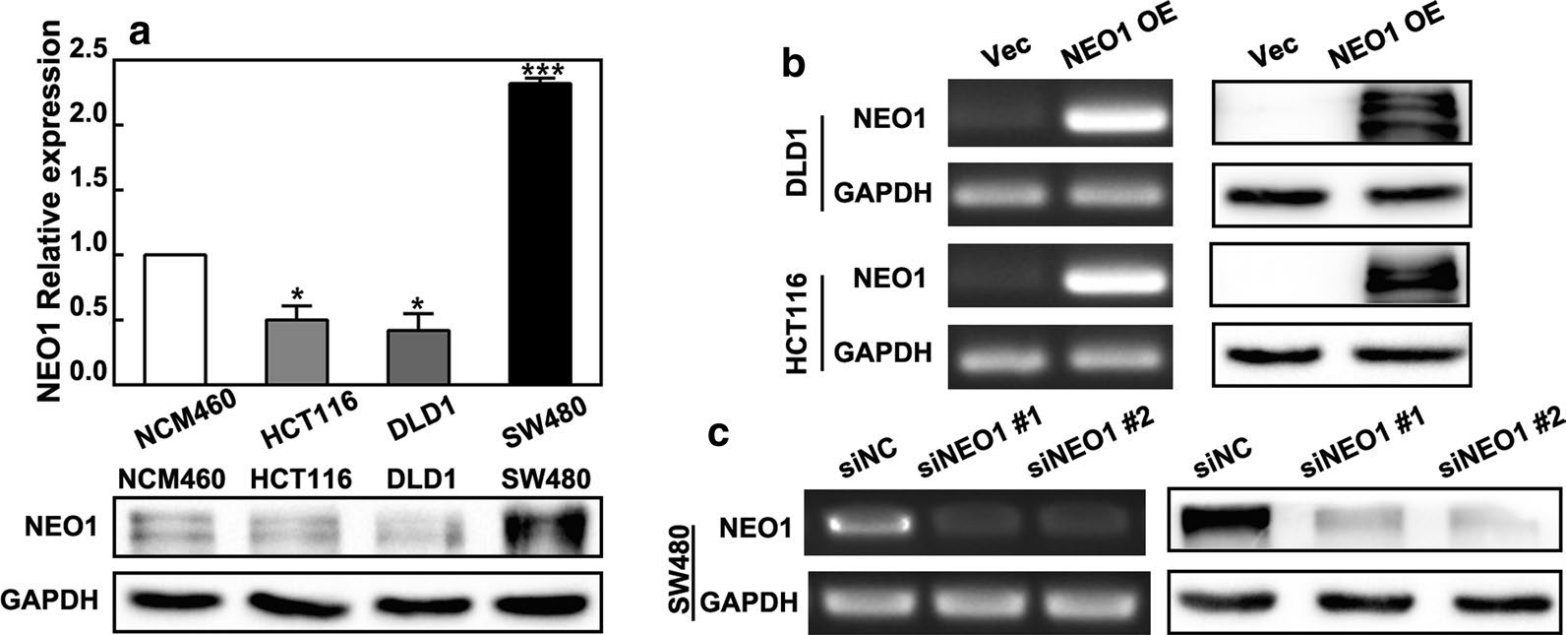
Transfection efficiency of NEO1 plasmids and siRNAs in CRC cells. **a** The mRNA and protein expression levels of NEO1 in three CRC cell lines were tested by real-time PCR and western blotting. **b** Reverse transcription PCR and western blotting analysis demonstrated that NEO1 was significantly over-expressed in DLD1 and HCT116 cells transfected with NEO1 plasmids (NEO1 OE) compared with empty plasmids (Vec). **c** Reverse transcription PCR and western blotting analysis demonstrated that NEO1 expression was reduced in SW480 cells transfected with NEO1 siRNAs (siNEO1 #1, #2) compared with negative control siRNAs (siNC). Data shown above are presented as the means ± s.e.m., n = 3; *P < 0.05, ***P < 0.001

**Fig. 5:**
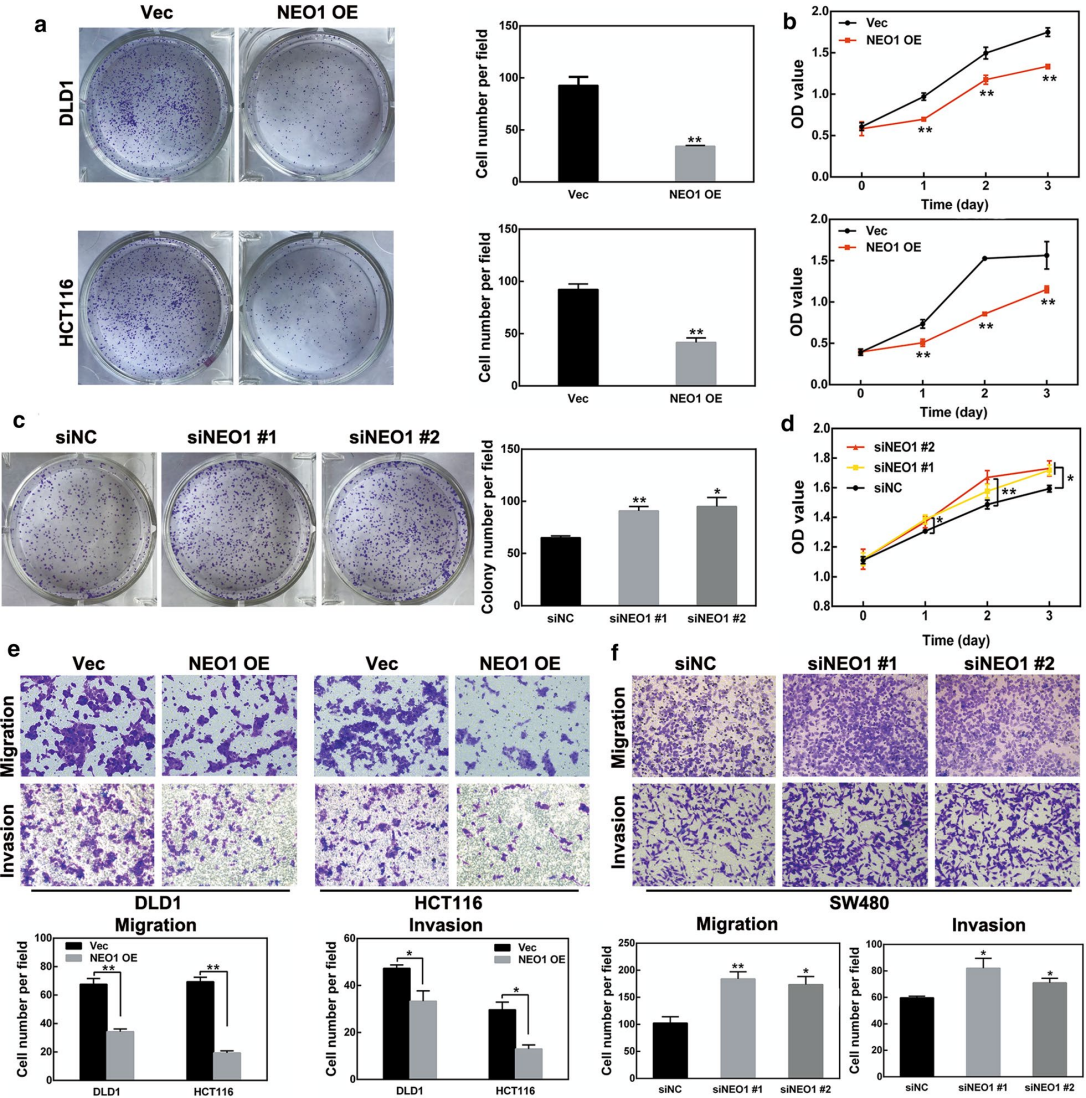
The biological role of NEO1 on CRC cell proliferation, migration and invasion. **a** Over-expression of NEO1 inhibited colony formation of DLD1 and HCT116 cells. **b** CCK8 assay showed that NEO1 over-expression inhibited DLD1 and HCT116 cell growth. **c**, **d** Knockdown of NEO1 promoted colony formation (**c**) and cell growth (**d**) of SW480. **e** Over-expression of NEO1 inhibited migration and invasion of DLD1 and HCT116 cells. **f** Knockdown of NEO1 induced migration and invasion of SW480 cells. Data shown above are presented as the means ± s.e.m., n = 3; *P < 0.05, **P < 0.01

The above results demonstrated that NEO1 expression was significantly down-regulated in human CRC tissues, and its expression was closely correlated with prognosis. In order to further investigate the biological role of NEO1 in CRC cells, colony formation, cell proliferation, migration and invasion assays were performed. Firstly, NEO1 expression levels in human colon cancer cell line HCT116, DLD1 and SW480 were examined while human colonic epithelial cell line NCM460 was used as a control. NEO1 showed relatively lower expression in DLD1 and HCT116 CRC cells and higher expression in SW480 CRC cells (Fig. 4a). Therefore, pCMV3-NEO1 plasmids were used to overexpress NEO1 expression in DLD1 and HCT116 cells (Fig. 4b). One the other hand, NEO1 expression in SW480 cells was silenced by two specific NEO1 siRNAs (siNEO1 #1, #2) (Fig. 4c). Our data showed that, in DLD1 and HCT116 cells, cell growth was distinctly inhibited in NEO1 overexpression cells compared with the control cells (Fig. 5a, b). However, NEO1 silencing induced cell growth in SW480 cells (Fig. 5c, d). Then, transwell assays showed that overexpression of NEO1 hindered the migratory and invasive ability of DLD1 and HCT116 cells (Fig. 5e). The opposite results were observed by NEO1 knockdown in SW480 cells (Fig. 5f). The above data demonstrated that NEO1 was involved in CRC progression by inhibiting cell growth, migration and invasion.

### Functional enrichment analysis of NEO1

**Fig. 6:**
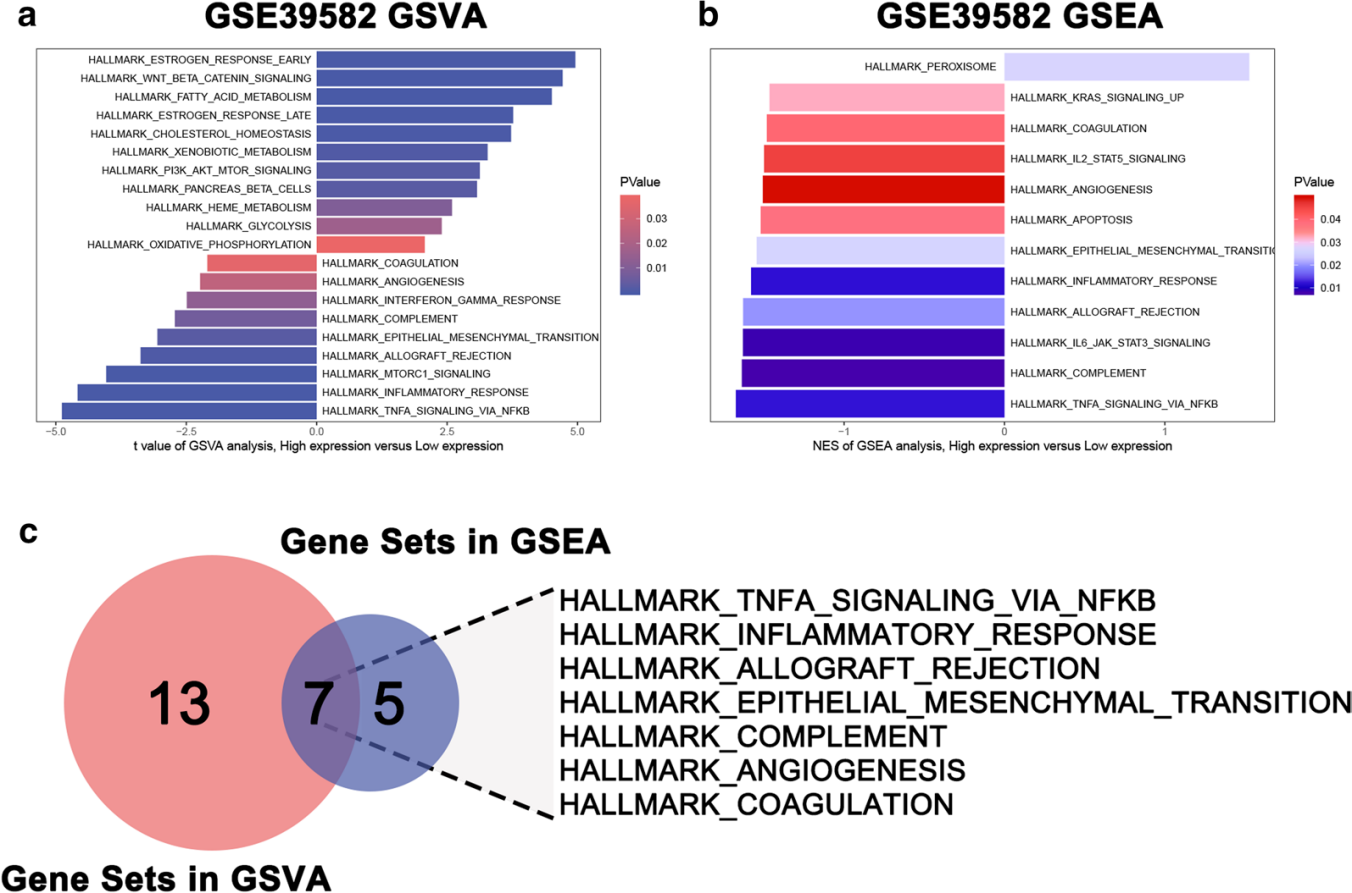
Gene set variation analysis (GSVA) and gene set enrichment analysis (GSEA) of NEO1 in GSE39582 dataset. **a** GSVA. **b** GSEA. **c** Common gene sets in GSVA and GSEA

To identify the distinct altered biological pathways between NEO1 high expression and low expression group, we performed the GSVA and GSEA by using GSE39582, which contained the most colon cancer samples. TNFα signaling via NFkB, inflammatory response, allograft rejection, epithelial mesenchymal transition (EMT), complement, angiogenesis and coagulation were particularly prominent between these two groups, both in GSVA analysis and GSEA analysis (Fig. 6).

## Discussion

Combined bioinformatics methods and CRC tissue validation, our data showed that NEO1 was down-regulated in CRC and the lower NEO1 expression level was found in advanced CRC samples. Furthermore, it was revealed that low expression of NEO1 had a poor clinical outcome, which suggested that NEO1 could be a prognosis marker for CRC patients.

In recent years, more and more attention has been paid to the heterogeneity of CRC. Inter-tumor heterogeneity and Intra-tumor heterogeneity are both challenges for CRC targeted treatment [6]. Inter-tumor heterogeneity means that CRC tissues in distinct patient present with vastly different genetic make‑ups, histopathological features and clinical behaviors. On the other hand, Intra-tumor heterogeneity refers to the genetic heterogeneity between cancer cells within a single tumor [6, 19]. Our data showed that CRC patients in different stage showed diverse NEO1 expression levels. What’s more, differential NEO1 expression levels induced distinct prognosis. These data demonstrated that NEO1 participated in the inter-tumor heterogeneity of CRC in a way.

Besides being the RGMa and netrin-1 receptor to mediate axonal guidance, NEO1 was proved to bind directly with bone morphogenetic proteins (BMPs). The study showed that BMP-2 binding to NEO1 led to activation of RhoA [20]. Also, the SHH/GLI pathway transcriptionally regulated NEO1 expression in nervous system [21]. As a homologue of the DCC gene [22], both DCC and NEO1 participated in ruling the balance between cell survival and death or between differentiation and de-differentiation, which led to an uncontrolled, excessive growth and potentially oncogenic phenotype [23, 24]. As a tumor suppressor, besides colorectal cancer, DCC defect was reported to contribute to the carcinogenesis in many other cancers, such as melanoma, breast, neuroblastoma and hematologic malignancies [23, 25]. The role of NEO1 in several cancer types has also been elucidated. It was reported that NEO1 expression was down-regulated in glioma and breast cancer and played as a tumor suppressor by inhibiting proliferation and inducing apoptosis [16, 26]. One the other hand, NEO1 was investigated to be overexpressed in gastric cancer and medulloblastoma, promoting cancer cell proliferation and motility [27, 28]. Therefore, the role of NEO1 was cancer type dependent (Additional file 1: Figure S1). Our present results showed that NEO1 played as a tumor suppressor gene in CRC, which was consistent with a recent study showing that NEO1 absence in Caco-2 CRC cells could induce a partial epithelial mesenchymal transition (EMT) [29].

The prognostication of CRC has been a prolonged topic all the time. Currently, it mainly depends on the Tumor Node Metastasis (TNM) staging system in clinical practice, which is not always performed well [30]. Therefore, researches of prognostic biomarkers emerged in endlessly. Several studies showed that SMAD4 could be as an efficient biomarker for CRC patient survival [31]. BRAF and KRAS mutations were also considered as valuable prognostic markers [31, 32]. BRAF and KRAS mutations could lead to the abnormal activation of Ras-Raf-MEK-ERK pathway, thus promoting the growth and proliferation of tumor cells [33]. These mutant patients are not only exposed to poor OS and DFS, but also insensitive for epidermal growth factor receptor (EGFR) antibody treatment [34, 35]. Though much efforts made in recent years, deeper researches are desiderated to identify markers that help doctors in estimating prognosis and treatment of CRC patients [36]. NEO1 was demonstrated closely connected with the OS and DFS in multiple datasets, which could be a new promising biomarker for CRC patients.

Functional pathway enrichment analysis revealed that NEO1 low expression samples were enriched in inflammation-related signaling pathways, EMT and angiogenesis. It’s widely recognized that inflammation and tumors are strictly interconnected and chronic inflammation can promote tumorigenesis [37, 38]. Previous studies have shown that NEO1 has a pivotal role during the onset of acute inflammation such as liver ischemia and reperfusion injury [39], acute peritoneal inflammation [40] and lung injury [41]. Moreover, a recent study demonstrated that NEO1 mediated local inflammation resolution and tissue regeneration processes. Knockdown of NEO1, on the one hand, induced apoptosis of neutrophils and then shortened their lifespan. The other is to facilitate clearance of human apoptotic PMNs or macrophages (MΦ) by activating eat-me and find-me signals and G protein–coupled receptors (GPCRs) [42]. However, whether and how NEO1 regulated inflammatory within tumor microenvironment of CRC remained unclear and needed further exploration. EMT is critical for tumor metastasis. Thus, the molecules who are closely related to EMT could be core biomarkers for early metastasis detection and novel targets for dedicated drug development. As an axon-guidance receptor, NEO1 influenced the direction and rate of cell motility and the direction and size of cell outgrowths such as lammellipodia, a key process of EMT [43]. It was validated that NEO1 absence in Caco-2 CRC cells could induce EMT by increasing Fibronectin 1 expression [29]. During angiogenesis, interactions between endothelial cells (EC) and associated perivascular cells are important in the regulation of vascular formation and stabilization [44]. Netrins, ligands of NEO1, are laminin-like secreted proteins, which have been proved to be involved in angiogenesis and blood vessel network formation [45, 46]. A study was shown that in endometriosis macrophage-derived Netrin-1 was vital for neuro-angiogenesis [47] while another shown that Netrin-4 was crucial for maintaining blood vessel by regulating endothelial and perivascular cells [48]. These studies indicated that the receptor NEO1 may be also involved in vasculogenesis of CRC, but needed further verification.

We have to mention some flaws in the present study. We revealed the possible role of NEO1 expression levels in inter-tumor heterogeneity, whether there were the genomic, non-genomic, stemness and microenvironment heterogeneity within a single CRC tissue needed further unveiling. Although the above results have predicted that NEO1 expression in CRC was closely associated with inflammation-related signaling pathways, EMT and angiogenesis, we haven’t done basic research to verify yet. Further research direction may focus on the effects of NEO1 on inflammation within tumor microenvironment. For example, the regulation of NEO1 on inflammatory cytokine release needs to be detected, and it would be interesting and meaningful to explore whether loss of NEO1 expression could induce immune escape by regulating T cells, macrophages or other immune cells. Furthermore, whether macrophage-derived Netrins could mediate CRC angiogenesis signaling through NEO1 is also worth exploring.

## Conclusion

In conclusion, our results identified a tumor suppressor NEO1 in CRC, which may serve as be a prognostic biomarker for CRC patients.

## Supplementary information

**Additional file 1: Figure S1.** The expression and role of NEO1 in different cancer types.

## Data Availability

The datasets used in this study are available from the corresponding author upon reasonable request.

## Abbreviations

CRC: Colorectal cancer
NEO1: Neogenin-1
DCC: Deleted in Colorectal Cancer
GSEA: Gene Set Enrichment Analysis
GSVA: Gene Set Variation Analysis
TCGA: The Cancer Genome Atlas
DEGs: Differentially expressed genes
GEO: Gene Expression Omnibus
CCK-8: Cell Counting Kit 8
COAD: Colon adenocarcinoma
READ: Rectal adenocarcinoma
CCAD: Cecum adenocarcinoma
CMAD: Colon mucinous adenocarcinoma
OS: Overall survival
DFS: Disease free survival
EMT: Epithelial mesenchymal transition

## Supplementary information

**Supplementary information** accompanies this paper at 10.1186/s12935-020-01604-1 .

## References

[1] Siegel RL, Miller KD, Fedewa SA, Ahnen DJ, Meester RGS, Barzi A (2017). Colorectal Cancer Statistics, 2017. CA Cancer J Clin.

[2] Wolf AMD, Fontham ETH, Church TR, Flowers CR, Guerra CE, LaMonte SJ (2018). Colorectal cancer screening for average-risk adults: 2018 guideline update from the American Cancer Society. CA Cancer J Clin.

[3] Kasi PM, Shahjehan F, Cochuyt JJ, Li Z, Colibaseanu DT, Merchea A (2019). Rising Proportion of Young Individuals With Rectal and Colon Cancer. Clin Colorectal Canc.

[4] Wang W, Kandimalla R, Huang H, Zhu L, Li Y, Gao F (2019). Molecular subtyping of colorectal cancer: Recent progress, new challenges and emerging opportunities. Semin Cancer Biol.

[5] Miller KD, Nogueira L, Mariotto AB, Rowland JH, Yabroff KR, Alfano CM (2019). Cancer treatment and survivorship statistics, 2019. CA Cancer J Clin.

[6] Punt CJA, Koopman M, Vermeulen L (2017). From tumour heterogeneity to advances in precision treatment of colorectal cancer. Nat Rev Clin Oncol.

[7] Qian F, Guo J, Jiang Z, Shen B (2018). Translational bioinformatics for cholangiocarcinoma: opportunities and challenges. Int J Biol Sci.

[8] Li R, Grimm SA, Mav D, Gu H, Djukovic D, Shah R (2018). Transcriptome and DNA methylome analysis in a mouse model of diet-induced obesity predicts increased risk of colorectal cancer. Cell Rep.

[9] Huang D, Sun W, Zhou Y, Li P, Chen F, Chen H (2018). Mutations of key driver genes in colorectal cancer progression and metastasis. Cancer Metast Rev.

[10] Okugawa Y, Toiyama Y, Toden S, Mitoma H, Nagasaka T, Tanaka K (2016). Clinical significance of SNORA42 as an oncogene and a prognostic biomarker in colorectal cancer. Gut.

[11] Shahjaman M, Kumar N, Ahmed MS, Begum A, Islam SMS, Mollah MNH (2017). Robust feature selection approach for patient classification using gene expression data. Bioinformation.

[12] Dalerba P, Sahoo D, Paik S, Guo X, Yothers G, Song N (2016). CDX2 as a prognostic biomarker in stage II and stage III colon cancer. New Engl J Med.

[13] Li VSW, Yuen ST, Chan TL, Yan HHN, Law WL, Yeung BHY (2009). Frequent inactivation of axon guidance molecule RGMA in human colon cancer through genetic and epigenetic mechanisms. Gastroenterology.

[14] Wilson NH, Key B (2006). Neogenin interacts with RGMa and Netrin-1 to guide axons within the embryonic vertebrate forebrain. Dev Biol.

[15] Villanueva AA, Puvogel S, Lois P, Munoz-Palma E, Ramirez OM, Lubieniecki F (2019). The Netrin-4/Laminin gamma1/Neogenin-1 complex mediates migration in SK-N-SH neuroblastoma cells. Cell Adh Migr.

[16] Wu X, Li Y, Wan X, Kayira TM, Cao R, Ju X (2012). Down-regulation of neogenin accelerated glioma progression through promoter methylation and its overexpression in shg-44 induced apoptosis. PLoS ONE.

[17] Lee JE, Kim HJ, Bae JY, Kim SW, Park J, Shin HJ (2005). Neogenin expression may be inversely correlated to the tumorigenicity of human breast cancer. BMC Cancer.

[18] Link B, Reichelt U, Schreiber M, Kaifi JT, Wachowiak R, Bogoevski D (2007). Prognostic implications of netrin-1 expression and its receptors in patients with adenocarcinoma of the pancreas. Ann Surg Oncol.

[19] Kyrochristos ID, Roukos DH (2019). Comprehensive intra-individual genomic and transcriptional heterogeneity: evidence-based colorectal cancer precision medicine. Cancer Treat Rev.

[20] Hagihara M, Endo M, Hata K, Higuchi C, Takaoka K, Yoshikawa H (2011). Neogenin, a receptor for bone morphogenetic proteins. J Biol Chem.

[21] Milla LA, Cortes CR, Hodar C, Onate MG, Cambiazo V, Burgess SM (2012). Yeast-based assay identifies novel Shh/Gli target genes in vertebrate development. BMC Genomics.

[22] Vielmetter J, Kayyem JE, Roman JM, Dreyer WJ (1994). Neogenin, an avian cell surface protein expressed during terminal neuronal differentiation, is closely related to the human tumor suppressor molecule deleted in colorectal cancer. J Cell Biol.

[23] Forrest CM, McNair K, Vincenten MCJ, Darlington LG, Stone TW (2016). Selective depletion of tumour suppressors deleted in colorectal cancer (DCC) and neogenin by environmental and endogenous serine proteases: linking diet and cancer. BMC Cancer.

[24] Matsunaga E, Tauszig-Delamasure S, Monnier PP, Mueller BK, Strittmatter SM, Mehlen P (2004). RGM and its receptor neogenin regulate neuronal survival. Nat Cell Biol.

[25] Boussouar A, Tortereau A, Manceau A, Paradisi A, Gadot N, Vial J (2020). Netrin-1 and Its receptor DCC are causally implicated in melanoma progression. Cancer Res.

[26] Zhang Q, Liang F, Ke Y, Huo Y, Li M, Li Y (2015). Overexpression of neogenin inhibits cell proliferation and induces apoptosis in human MDA-MB-231 breast carcinoma cells. Oncol Rep.

[27] Kim SJ, Wang YG, Lee HW, Kang HG, La SH, Choi IJ (2014). Up-regulation of neogenin-1 increases cell proliferation and motility in gastric cancer. Oncotarget.

[28] Milla LA, Arros A, Espinoza N, Remke M, Kool M, Taylor MD (2014). Neogenin1 is a Sonic Hedgehog target in medulloblastoma and is necessary for cell cycle progression. Int J Cancer.

[29] Chaturvedi V, Fournier-Level A, Cooper HM, Murray MJ (2019). Loss of Neogenin1 in human colorectal carcinoma cells causes a partial EMT and wound-healing response. Sci Rep.

[30] Bramsen JB, Rasmussen MH, Ongen H, Mattesen TB, Ørntoft MW, Árnadóttir SS (2017). Molecular-subtype-specific biomarkers improve prediction of prognosis in colorectal cancer. Cell Rep.

[31] Voorneveld PW, Jacobs RJ, Kodach LL, Hardwick JCH (2015). A Meta-analysis of SMAD4 immunohistochemistry as a prognostic marker in colorectal cancer. Transl Oncol.

[32] de Cuba EMV, Snaebjornsson P, Heideman DAM, van Grieken NCT, Bosch LJW, Fijneman RJA (2016). Prognostic value of BRAF and KRAS mutation status in stage II and III microsatellite instable colon cancers. Int J Cancer.

[33] Jones GG, Del Río IB, Sari S, Sekerim A, Young LC, Hartig N (2019). SHOC2 phosphatase-dependent RAF dimerization mediates resistance to MEK inhibition in RAS-mutant cancers. Nat Commun.

[34] Misale S, Yaeger R, Hobor S, Scala E, Janakiraman M, Liska D (2012). Emergence of KRAS mutations and acquired resistance to anti-EGFR therapy in colorectal cancer. Nature.

[35] Brudvik KW, Kopetz SE, Li L, Conrad C, Aloia TA, Vauthey JN (2015). Meta-analysis of KRAS mutations and survival after resection of colorectal liver metastases. Brit J Surg.

[36] Duan L, Yang W, Wang X, Zhou W, Zhang Y, Liu J (2019). Advances in prognostic markers for colorectal cancer. Expert Rev Mol Diagn.

[37] Dominguez C, David JM, Palena C (2017). Epithelial-mesenchymal transition and inflammation at the site of the primary tumor. Semin Cancer Biol.

[38] Francescone R, Hou V, Grivennikov SI (2014). Microbiome, Inflammation, and Cancer. Cancer J.

[39] Schlegel M, Granja T, Kaiser S, Körner A, Henes J, König K (2014). Inhibition of neogenin dampens hepatic ischemia-reperfusion injury. Crit Care Med.

[40] König K, Gatidou D, Granja T, Meier J, Rosenberger P, Mirakaj V (2012). The axonal guidance receptor neogenin promotes acute inflammation. PLoS ONE.

[41] Mirakaj V, Jennewein C, König K, Granja T, Rosenberger P (2011). The guidance receptor neogenin promotes pulmonary inflammation during lung injury. Faseb J.

[42] Schlegel M, Korner A, Kaussen T, Knausberg U, Gerber C, Hansmann G (2018). Inhibition of neogenin fosters resolution of inflammation and tissue regeneration. J Clin Invest.

[43] Stone TW (2020). Dependence and guidance receptors—DCC and neogenin—In Partial EMT and the Actions of Serine Proteases. Front Oncol.

[44] Teichert M, Milde L, Holm A, Stanicek L, Gengenbacher N, Savant S (2017). Pericyte-expressed Tie2 controls angiogenesis and vessel maturation. Nat Commun.

[45] Huyghe A, Furlan G, Ozmadenci D, Galonska C, Charlton J, Gaume X (2020). Netrin-1 promotes naive pluripotency through Neo1 and Unc5b co-regulation of Wnt and MAPK signalling. Nat Cell Biol.

[46] Sung P, Rama N, Imbach J, Fiore S, Ducarouge B, Neves D (2019). Cancer-associated fibroblasts produce netrin-1 to control cancer cell plasticity. Cancer Res.

[47] Guo X, Ding S, Li T, Wang J, Yu Q, Zhu L (2020). Macrophage-derived netrin-1 is critical for neuroangiogenesis in endometriosis. Int J Biol Macromol.

[48] Lejmi E, Bouras I, Camelo S, Roumieux M, Minet N, Leré-Déan C (2014). Netrin-4 promotes mural cell adhesion and recruitment to endothelial cells. Vasc Cell.

