# Supplementary figures and images for: Identification of NEO1 as a prognostic biomarker and its effects on the progression of colorectal cancer
Source: Cancer Cell Int. 2020 Oct 17;20:510. doi: 10.1186/s12935-020-01604-1 (PMC7568410; doi:10.1186/s12935-020-01604-1)

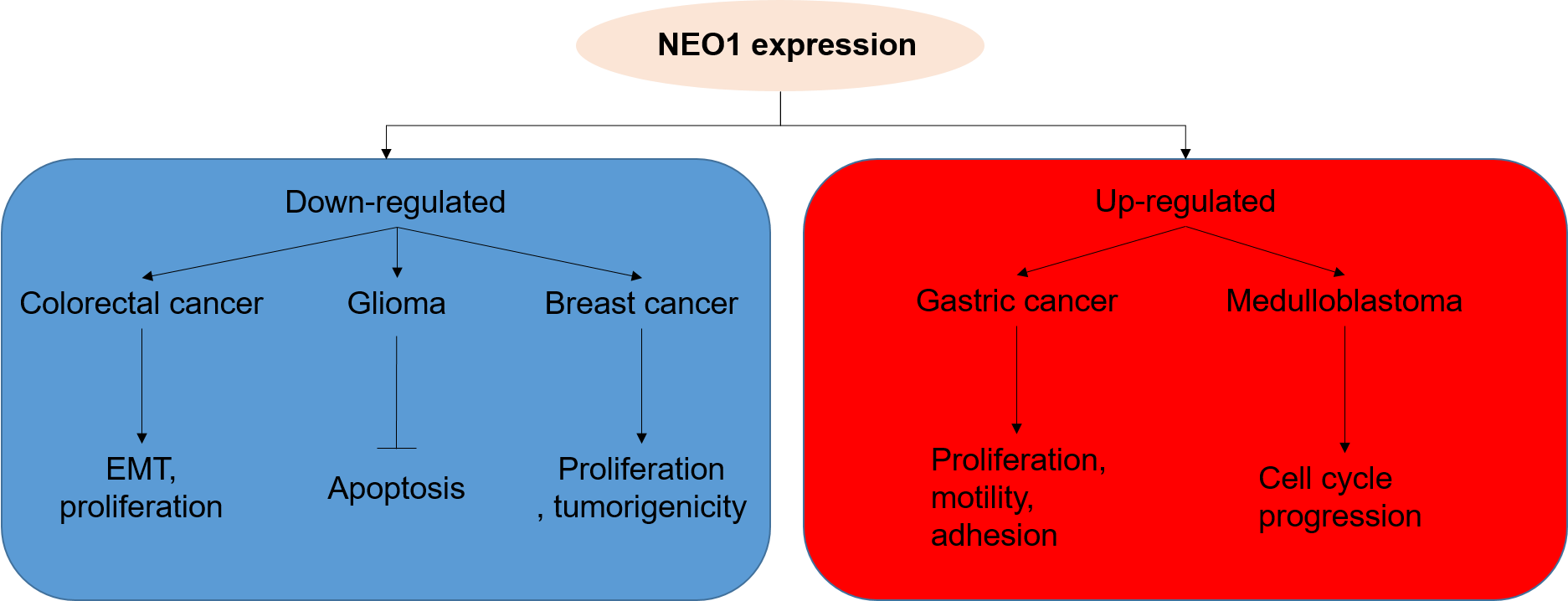

Supplement: Supplementary file 1 — Additional file 1: Figure S1. The expression and role of NEO1 in different cancer types. [file 12935_2020_1604_MOESM1_ESM.tif]
